# New Method of Treating Hemorrhoidal Tumors

**Published:** 1877-07

**Authors:** 


A NEW method of treating hemorrhoidal tumors is brought to the notice of the profession through *The Journal of Materia Medica*; i.e. by carbolic acid, with the addition of olive oil in the following proportions: carbolic acid ℨij., olive oil ℨi., 4 or 5 drops, to be injected into the tumor with a hypodermic syringe. The operation is said to be almost completely painless, the tumor at the point of insertion turning white. The inflammation is slight, suppurative action being established in a few days. When one tumor is treated at a time the patient can with ease follow his usual avocations. Fissures also heal rapidly when treated once or twice a week with the same solution.

